# The Ternary Heterostructures of BiOBr/Ultrathin g-C_3_N_4_/Black Phosphorous Quantum Dot Composites for Photodegradation of Tetracycline

**DOI:** 10.3390/polym10101118

**Published:** 2018-10-09

**Authors:** Tianhao Jiang, Chaoqun Shang, Qingguo Meng, Mingliang Jin, Hua Liao, Ming Li, Zhihong Chen, Mingzhe Yuan, Xin Wang, Guofu Zhou

**Affiliations:** 1 International Academy of Optoelectronics at Zhaoqing, South China Normal University, Guangzhou 510631, China; tianhao.jiang@ecs-scnu.org (T.J.); chaoqun.shang@ecs-scnu.org (C.S.); jinml@scnu.edu.cn (M.J.); guofu.zhou@m.scnu.edu.cn (G.Z.); 2 Shenyang Institute of Automation, Chinese Academy of Sciences Guangzhou, Guangzhou 510631, China; qgmeng@gmail.com (Q.M.); mzyuan@sia.cn (M.Y.); 3 National Center for International Research on Green Optoelectronics, South China Normal University, Guangzhou 510631, China; 4 Institute of Solar Energy, Yunnan Normal University, Kunming 650500, China; liaohua8@ynnu.edu.cn (H.L.); lmllldy@126.com (M.L.)

**Keywords:** BiOBr, ultrathin g-C_3_N_4_, black phosphorous quantum dots, photodegradation, antibiotics

## Abstract

Herein, we synthesized BiOBr/ultrathin g-C_3_N_4_/ternary heterostructures modified with black phosphorous quantum dots using a simple water bath heating and sonication method. The ternary heterostructure was then used for the photocatalytic degradation of tetracycline in visible light, with an efficiency as high as 92% after 3 h of irradiation. Thus, the photodegradation efficiency is greatly improved compared to that of ultrathin g-C_3_N_4_, BiOBr, and black phosphorous quantum dots alone. The synthesized ternary heterostructure improves the charge separation efficiency, thus increasing the photodegradation efficiency. This work provides a new and efficient method for the degradation of antibiotics in the environment.

## 1. Introduction

Given the increasing concern regarding environmental pollution due to antibiotics and growing awareness of anti-microbial resistance, the demand for new methods for antibiotic environmental remediation is increasingly urgent. The overuse of broad-spectrum antibiotics, such as tetracycline (TC) [1], has led to widespread aquatic system pollution, disturbing the ecological balance and posing a major threat to human health [2,3,4,5]. Therefore, there is a need for efficient, low cost, and sustainable methods for water-system environmental remediation. The use of inexhaustible solar energy resources to degrade antibiotics through the photocatalytic action of semiconductors is undoubtedly a worthwhile application, with considerable industrialization potential. Nevertheless, a semiconductor photocatalyst that can be used to degrade antibiotics at low cost, with an easy production method, and powerful functions remains to be developed [6,7].

C_3_N_4_ is a lamellar, organic, non-metallic polymeric material formed from nitrogen and carbon in accordance with SP_2_ hybridization [8,9]. Its appropriate bandgap width (2.7 ev) and high chemical stability make g-C_3_N_4_ one of the most suitable photodegradable antibiotic catalysts [10,11,12]. Unfortunately, g-C_3_N_4_ only has high photocatalytic activity at a wavelength range above 460 nm. Therefore, there is a need to increase the photocatalytic activity range of g-C_3_N_4_ to achieve a visible light response and higher efficiency. This can be achieved through the production of new photodegradation catalysts based on g-C_3_N_4_. Several methods have been proposed to effectively enhance the activity of g-C_3_N_4_, for example, by reducing the thickness of g-C_3_N_4_ nanosheets and increasing their specific surface area or copolymerizing g-C_3_N_4_ with other semiconductors to form heterojunctions to accelerate the separation of electron (e^−^)–hole (h^+^) pairs and increase the lifetime of the photo-generated carriers [13,14,15].

Herein, we first synthesized an ultrathin g-C_3_N_4_ nanosheet (UCN) by blowing dicyandiamide with ammonium chloride to increase its specific surface area [16,17,18]; this would achieve an increase in surface area for suitable antibiotic interactions. Secondly, we coupled the UCN with an inorganic composite photocatalyst BiOBr. BiOBr has a highly suitable photocatalytic redox bandgap (2.75 eV) under visible light and has been widely used in the photocatalytic degradation of dyes, antibiotics, and other substances [19,20,21,22,23]. UCNs doped with BiOBr through a simple stirring and hydrothermal procedure to form a heterojunction to promote the separation of e^−^–h^+^ pairs, have shown an enhanced catalytic degradation efficiency of UCN under visible light [24,25]. Nevertheless, the achieved degradation efficiencies are not yet sufficient for adequate antibiotic degradation [26,27,28].

Recently black phosphorus has received considerable attention from the scientific community because of its excellent performance. It is a two-dimensional atomic material similar to graphene, with an energy bandgap that can be regulated by the number of layers, and with a wavelength absorbance ranging from visible light to the infrared [29]. In addition, black phosphorus is a direct energy gap semiconductor that converts electronic signals into optical signals. Black phosphorus quantum dots (BPQDs) have been recently developed. Due to quantum definition and edge effects, BPQDs have excellent optical and electrical properties [30,31,32]. Recently, BPQDs were introduced as a hole-migration *co*-catalyst for visible-light-driven water splitting, and it has been demonstrated that the performance could be improved by loading BPQDs on the layered g-CN due to their unique optical and electrical properties [33].

In order to further improve its photocatalytic performance, we developed a ternary heterostructure of BiOBr/UCN decorated with BPQDs for the photocatalytic degradation of TC with the required degradation efficiency.

## 2. Experimentals 

### 2.1. Materials

Ammonium chloride, dicyandiamide (analytical grade), bismuth nitrate pentahydrate (Bi(NO_3_)_3_·5H_2_O, analytical grade), potassium bromide (analytical grade), and TC (analytical pure and used without further purification) were purchased from Shanghai Aladdin Industrial Corporation (Shanghai, China). Anhydrous ethanol, dimethylformamide (Shanghai Aladdin Industrial Corporation, Shanghai, China), and black phosphorus were obtained from Nanjing Materials Technology Companies (Nanjing, China).

### 2.2. Preparation of UCNs

Ammonium chloride (20 g) and dicyandiamide (4 g) were dissolved in a suitable amount of water and stirred at 80 °C until the mixture dried out. The resulting material was then thoroughly ground and placed in a muffle furnace (Hefei Kejing Materials Technology Co., LTD., Hefei, China) and heated to 550 °C at a ramp rate of 3 °C per minute for 4 h. Finally, the product was fully ground, producing ultrathin layer UCNs [16,17,18].

### 2.3. Preparation of a BiOBr/UCN (7:3) Photocatalytic System

First, 0.273 g of potassium bromide was dissolved in a suitable amount of deionized water and sonicated for 5 min; 0.3 g of UCNs were weighed, mixed into the above solution, and sonicated for 5 min. Finally, 1.113 g of bismuth nitrate pentahydrate was added to the above mixture and sonicated for 5 min. The above mixed solution was placed in a water bath at 80 °C for 3 h, and finally dried in a drying oven (Shanghai Boxun Industry and Commerce Co., LTD., Medical Equipment Factory, Shanghai, China) to obtain the BiOBr/UCN photocatalytic system [17,22,26,27,28].

### 2.4. Preparation of BiOBr/UCN/BPQDs Ternary Structure

**Figure 1:**
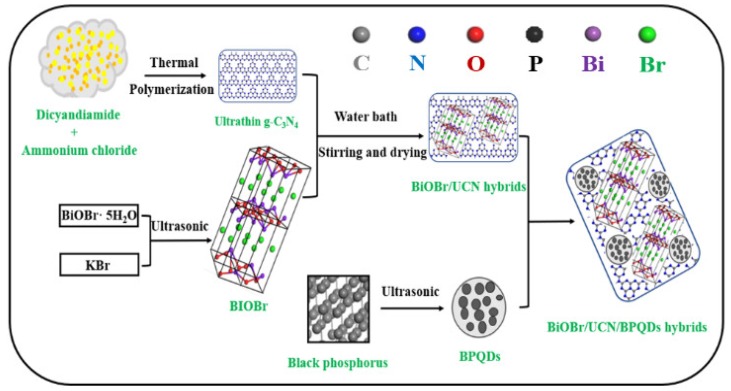
Schematic route of the production of BiOBr/UCN/BPQDs hybrids.

Black phosphorus is a graphite-like material with excellent electron transfer properties. High-quality, low-layer black phosphorous nanosheets can be stripped by high-intensity ultrasound. Therefore, we measured 10 mg of black phosphorus and placed it in a volumetric flask (Hua Ou Industry, Yancheng, China) containing 10 mL of dimethylformamide, sonicated the mixture for 4 h using an ultrasonic cell crusher (Ningbo Scientz Biotechnology Co., LTD., Ningbo, China); and then 1 mg/mL of BPQD dispersions were prepared. (It is worth noting that before ultrasound, 10 mg of black phosphorus was pressed on the foam copper network (Shenzhen Wang Xing Tape Co., LTD., Shenzhen, China) as the positive electrode of the battery, and the sodium electrode as the negative electrode of the battery. Under the 3v voltage window, the constant current charge and discharge lasted for 2 weeks [31].) Next, the BPQDs were mixed with the BiOBr/UCN suspension and stirred for 4 h, and then BiOBr/UCN/BPQDs hybrids were collected by centrifugation. Figure 1 shows the general production process of BiOBr/UCN/BPQDs hybrids [29,30,31,32,33,34].

## 3. Results and Discussion

### 3.1. Physicochemical Properties of BiOBr/UCN/BPQDs Heterojunctions

#### 3.1.1. SEM and TEM Analysis

**Figure 2:**
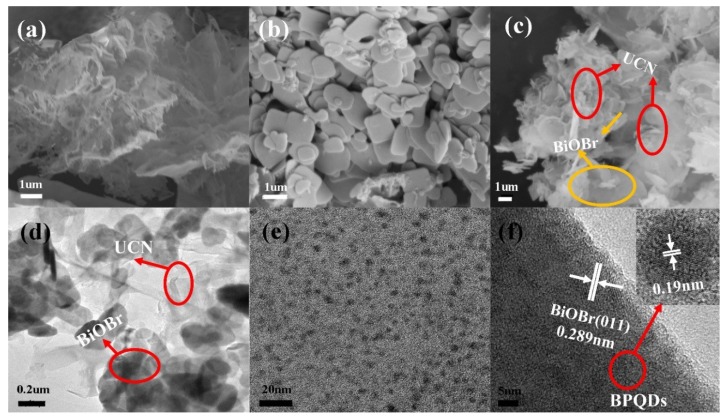
SEM images of (**a**) ultrathin g-C_3_N_4_ nanosheets, (**b**) BiOBr nanosheets, and (**c**) BiOBr/UCN/BPQDs ternary heterostructure. TEM images of (**d**) BiOBr/UCN, (**e**) BPQDs, and (**f**) BiOBr/UCN/BPQDs ternary heterostructure.

SEM and TEM images (Figure 2) proved the formation of well-compounded, BiOBr/UCN/BPQDs ternary heterostructures. The observed structure of the prepared UCNs (Figure 2a) shows an ultra-thin, transparent, multi-layered structure that has a larger contact area per unit mass than a normal bulk structure, and thus, has a higher catalytic efficiency. The BiOBr material was composed of a dense array of nanosheets (Figure 2b). Following UCN polymerization on the BiOBr nanosheet and BPQD decoration, an ultra-thin layer g-C_3_N_4_ was observed (Figure 2c), and confirmed by TEM (Figure 2d), wherein ultra-thin, transparent lamellar g-C_3_N_4_ coated the dark opaque BiOBr flakes. Further, the densely packed BPQDs were evenly distributed on the surface of the BiOBr/UCN composite (Figure 2e). High-resolution TEM showed the lattice spacing of BPQDs attached to BiOBr/UCN as 0.19 nm and the lattice spacing of BiOBr as 0.289 nm, corresponding to the (011) crystal plane (Figure 2f) [31]. The intimate contact of this ternary heterostructure may significantly accelerate the separation of e^–^–h^+^ pairs, thereby further enhancing photocatalytic performance [16,27,28,30].

#### 3.1.2. X-ray Diffraction (XRD) Analysis

**Figure 3:**
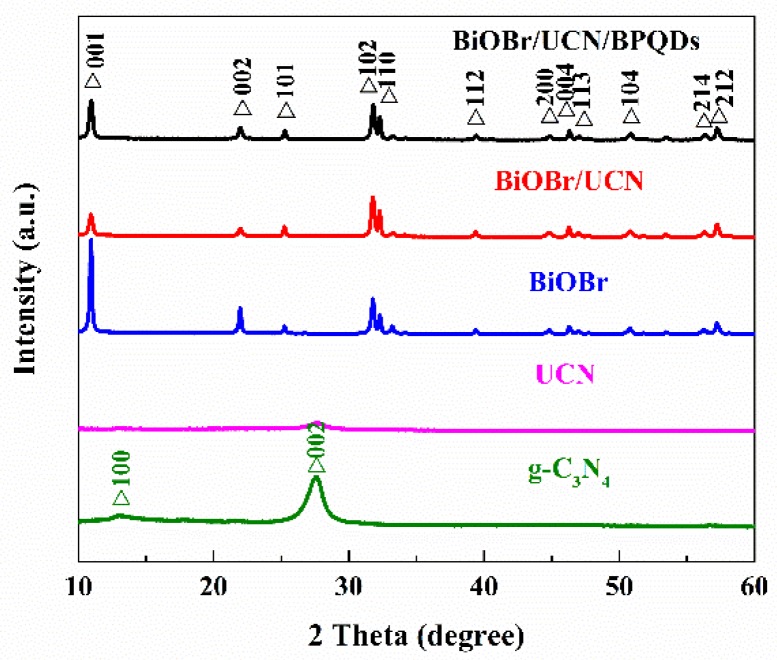
XRD of pure g-C_3_N_4_, UCN, pure BiOBr, BiOBr/UCN, and BiOBr/UCN/BPQDs.

XRD was used to study the phase structure of BiOBr/UCN/BPQDs composite catalysts. The corresponding XRD patterns of g-C_3_N_4_, UCN, BiOBr, BiOBr/UCN, and BiOBr/UCN/BPQDs were obtained (Figure 3). Compared with g-C_3_N_4_, the diffraction peaks of UCN at 13.27° (100) and 27.51° (002) were obviously weakened, indicating a change in the structural properties of UCN. The main diffraction peaks of pure BiOBr are located at 10.94°, 21.99°, 25.24°, 31.78°, 32.29°, 39.38°, 44.81°, 46.29°, 46.98°, 50.79°, 56.29°, and 57.20°, belonging to the (001), (002), (101), (102), (110), (112), (200), (004), (113), (104), (212), and (214) planes of BiOBr crystal diffraction square phase (JCPDS No. 78-0348). Following UCN and BiOBr doping at a mass ratio of 3:7, the peaks of planes (100) and (002) disappeared, the diffraction peak of BiOBr at the crystal plane (001) was weakened. Moreover, the (001) crystal phase peak observed for the BiOBr/UCN composite increased slightly in intensity following doping by BPQDs. Nevertheless, following the addition of BPQDs, the black phosphorus peaks did not appear, likely due to the minimal content [17,27,35].

#### 3.1.3. FT-IR Analysis

**Figure 4:**
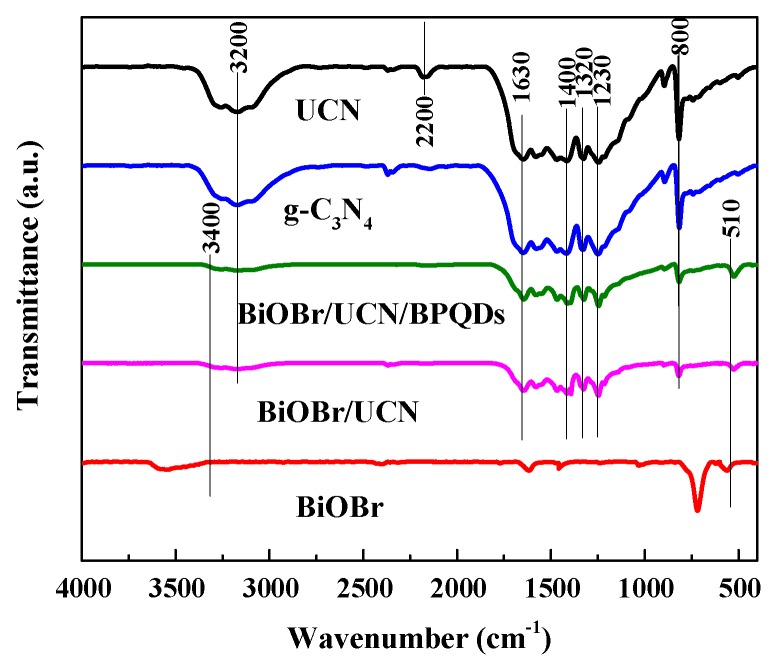
FT-IR analysis of pure g-C_3_N_4_, UCN, pure BiOBr, BiOBr/UCN, and BiOBr/UCN/BPQDs.

In order to further confirm the formation of BiOBr/UCN/BPQDs composites, the pure g-C_3_N_4_, UCN, pure BiOBr, BiOBr/UCN, and BiOBr/UCN/BPQDs were assessed by FT-IR spectroscopy (Figure 4). In the pure BiOBr sample, the bandwidth centered around 1630 cm^−1^ was assigned to the bending vibration stretching of –OH, and those at 510, 3400 cm^−1^ were attributed to the absorption mode of the Bi–O bond. Samples with UCN showed absorption bands between 1150 and 1700 cm^−1^; the band near 1630 cm^−1^ was attributed to the stretching of the C–N bond, whereas those at 1230, 1320, and 1400 cm^−1^ correspond to the curvature of the C–N bond. At approximately 2200 cm^−1^, UCN showed a small characteristic peak, distinct from pure g-C_3_N_4_. The characteristic pattern of s-triazine was observed near 800 cm^−1^, and was also observed in BiOBr/UCN and BiOBr/UCN/BPQDs, indicating that the structure of UCN was maintained during doping with BiOBr and BPQDs.

Thus, XRD, FT-IR, and TEM results showed that UCN was efficiently doped with BiOBr and BPQDs [28].

#### 3.1.4. XPS Analysis

**Figure 5:**
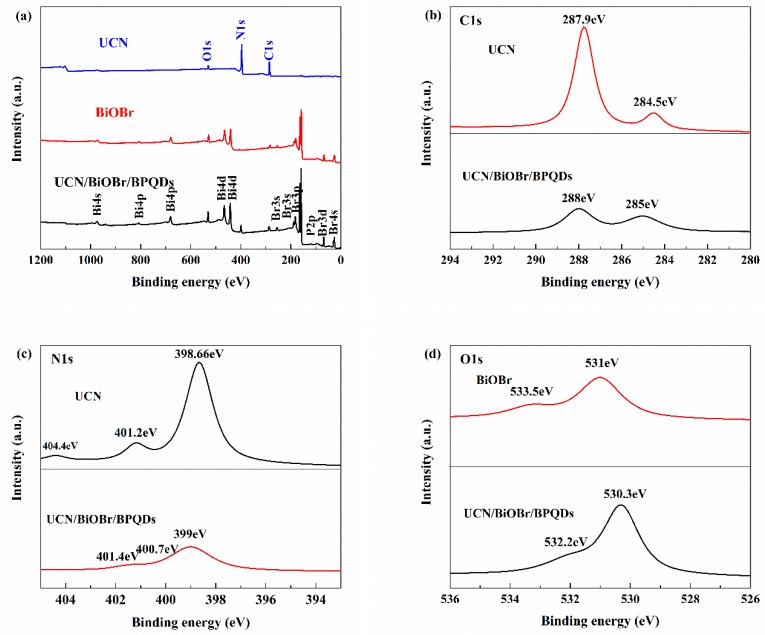

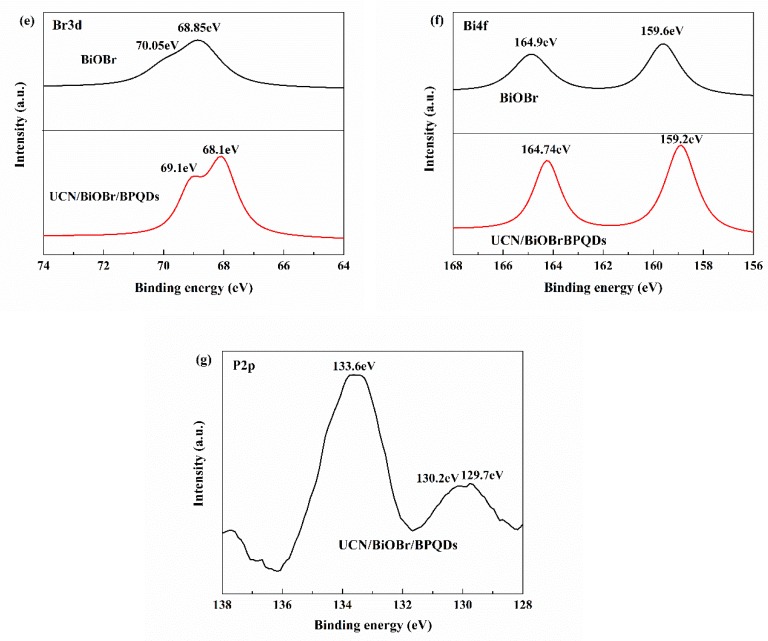
XPS of UCN, BiOBr, and BiOBr/UCN/BPQDs. (**a**) survey; (**b**) C1s; (**c**) N1s; (**d**) O1s; (**e**) Br3d; (**f**) Bi4f; (**g**) P2p.

XPS was used to further assess the chemical state of the constituent elements (Figure 5). Obviously, BiOBr/UCN/BPQDs were composed of C, N, O, Bi, Br, and P [36]. UCN’s N1s, C1s, with a small amount of O1s spectra, spectra of Bi4s, Bi4p, Bi4d, O1s, Br3s, Br3d, Br3p, Br4s of BiOBr, the N1s, C1s, Bi4s, Bi4p, Bi4d, O1s, Br3s, Br3d, Br3p, Br4s and P2p spectra of BiOBr/UCN/BPQDs were observed (Figure 5a). The C1s spectra of UCN and BiOBr/UCN/BPQDs, with peaks at 287.9 and 288 eV, indicate the presence of C–N–C bonds, whereas peaks at 284.5 and 285 eV correspond to surface adventitious carbon (Figure 5b). The N1s spectrum showed main peaks at 398.66 and 399 eV, indicating the formation of C=N–C bonds between nitrogen and carbon atoms, which exist as SP^2^ hybrids (Figure 5c). The other two small peaks (400.7, 401.4 eV), correspond to (N–(C)_3_) and N–H groups, respectively. The weak peak at 404.4 eV indicates that the CN atom was hybridized in the form of π bond. The O1s spectra of BiOBr and BiOBr/UCN/BPQDs (Figure 5d) showed that, due to the presence of Bi–O bonds, BiOBr had a peak at 531 eV, while that of BiOBr/UCN/BPQDs appeared at 530.3 eV, showing lower binding energy. Correspondingly, the peaks at 164.9 and 159.6 eV (Figure 5e) correspond to the two spin-orbital components of Bi4f^5/2^ and Bi4f^7/2^ in BiOBr. When combined with UCN and BPQDs, these two peaks were converted to 164.74 and 159.2 eV, respectively. Similarly, the peaks for Br3d^5/2^ and Br3d^3/2^ in pure BiOBr and BiOBr/UCN/BPQDs were 68.85, 70.05, and 68.1, 69.1eV, respectively (Figure 5f). Finally, the crystal characteristic peaks of 2P^3/2^ and 2P^1/2^ for BPQDs were observed at 129.7 and 130.2 eV, respectively, while that at 133.6 eV corresponded to the phosphorus oxide (PO_x_) peak (Figure 5g) [27,29,30,31,36].

#### 3.1.5. Photoluminescence Spectra and Optical Performance Analysis

**Figure 6:**
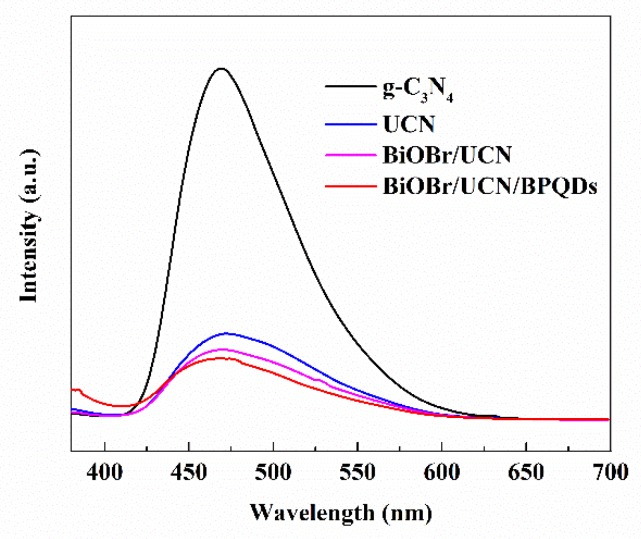
Photoluminescence spectra of pure g-C_3_N_4_, UCN, BiOBr/UCN, and BiOBr/UCN/BPQDs.

Photoluminescence spectra were used to analyze the migration and recombination of photoelectron (e^−^)–h^+^ pairs in the composites. The main emission peak was centered at 470.6 nm, with pure g-C_3_N_4_ showing the highest peak, followed by that of UCN, BiOBr/UCN, and finally BiOBr UCN/BPQDs (Figure 6); these results show that the compound probabilities of their photo-generated carriers decrease in turn [4].

**Figure 7:**
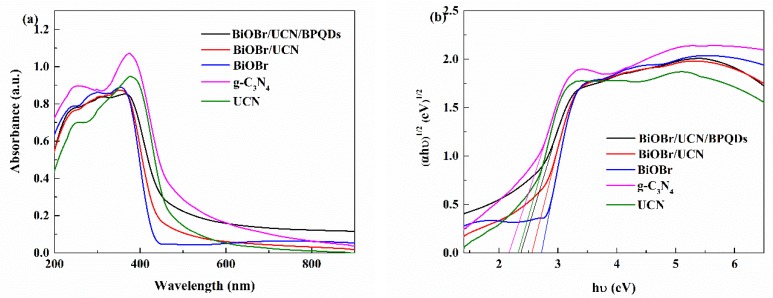
(**a**) UV-visible diffuse reflectance spectra of samples, (**b**) (αhν)^1/2^ for photon energy hν curve.

The optical properties of g-C_3_N_4_, UCN, BiOBr, BiOBr/UCN, and BiOBr/UCN/BPQDs were assessed by UV-visible diffuse reflectance spectroscopy (Figure 7). The absorption edge of UCN showed an obvious blue shift compared with that of g-C_3_N_4_ (Figure 7a). Therefore, based on the data in Figure 7b and using (αhν)^1/2^(eV)^1/2^ and photon energy hν (eV) to roughly calculate the band gap of UCN, a value of 2.4 eV was obtained, which is larger than that for pure g-C_3_N_4_ (2.2 eV). The light absorption edge of BiOBr was located near 430 nm, with a subsequent calculated band gap of 2.7 eV. Following the formation of a composite, the absorption edges of UCN and BPQDs fell between 430 and 500 nm, and increased sequentially, indicating that these composites are sensitive to and can work well under visible light [21,23].

### 3.2. Photocatalytic Performance of BiOBr/UCN/BPQDs Heterostructures

**Figure 8:**
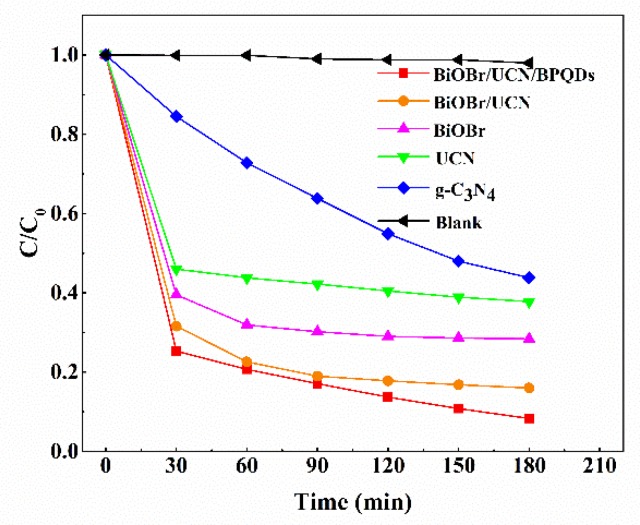
Degradation of tetracycline by catalyst under visible light.

The catalytic activity of these five photocatalysts was measured according to the degradation of TC in water by visible light. For a typical photocatalytic measurement, 0.25 g of the photocatalyst was evenly dispersed in the solution of TC (100 mL, initial concentrations of which were all 30 mg/L) through ultrasonication. Experiments were conducted in a 200 mL reactor, associated with a cooling water circulation system. Temperature of the system was controlled at 25 °C throughout the reaction. After reaching a state of equilibrium (30 min), the mixture was subjected to the xenon lamp (Profect Light, CEL-HXF300, AM 1.5 output, Beijing Zhongjiao Jinyuan Technology Co., LTD., Beijing, China) with 420 nm-cut filter. Suspension (4 mL) was sampled at given time intervals. Magnetic stirring was conducted before (the adsorption/desorption stage) and during the photocatalytic reactions. After centrifugation, the residual concentration of TC was monitored by the UV-vis spectrophotometer (METASH UV-8000A, SHJH (Group) Company, Shanghai, China). BiOBr/UCN/BPQDs composites showed the best photodegradation efficiency, with a degradation efficiency of up to 92% within 3 h (Figure 8) [15,20,24,25,26]. The catalytic performance of BiOBr/UCN composites was slightly lower, reaching 84%, whereas that of pure BiOBr reached 71.6%. The degradation efficiency of UCN (37.8%) was significantly higher than that of g-C_3_N_4_ (43.8%) [7,37,38,39].

**Figure 9:**
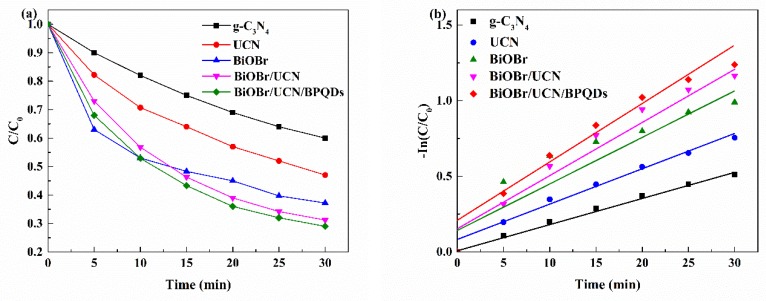
(**a**) Degradation of tetracycline within 30 min, and (**b**) corresponding kinetic equations.

The kinetics of photodegradation of TC were then assessed at 30 min and at every 5 min thereafter to obtain the corresponding kinetic equation. The degradation curve of TC at 30 min (Figure 9a) allowed the plotting of the corresponding kinetic equation (Figure 9b), calculated by Equation (1).

In(*C*_0_/*C*) = *Kt* (1)

where, *K* is the pseudo first-order rate constant, *C*_0_ is the initial concentration of TC solution, *C* is the concentration of TC at times *t*.

The *K* values corresponding to BiOBr/UCN/BPQDs, BiOBr/UCN, BiOBr, UCN, and pure g-C_3_N_4_ were 0.041, 0.038, 0.032, 0.025, and 0.017 min^−1^, respectively. Therefore, these results also indicated that had the best photocatalytic properties. 

**Figure 10:**
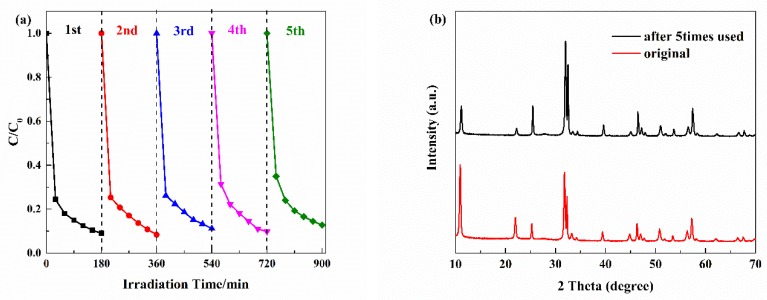
(**a**) Five cycles of experiments using samples with the best catalytic effect; (**b**) XRD patterns before and after the five cycles.

In order to assess the stability of the as-prepared BiOBr/UCN/BPQDs, we performed recycles of the photocatalytic experiments and XRD. The catalytic performance of BiOBr/UCN/BPQDs did not decrease significantly (Figure 10a), and the composite was shown to have good structural stability (Figure 10b) [4,22].

### 3.3. Possible Photocatalytic Mechanism of BiOBr/UCN/BPQDs

**Figure 11:**
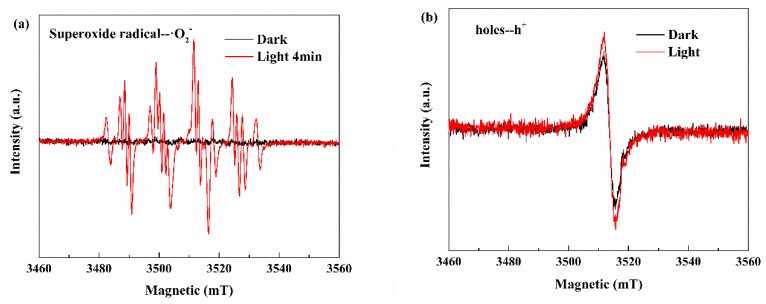

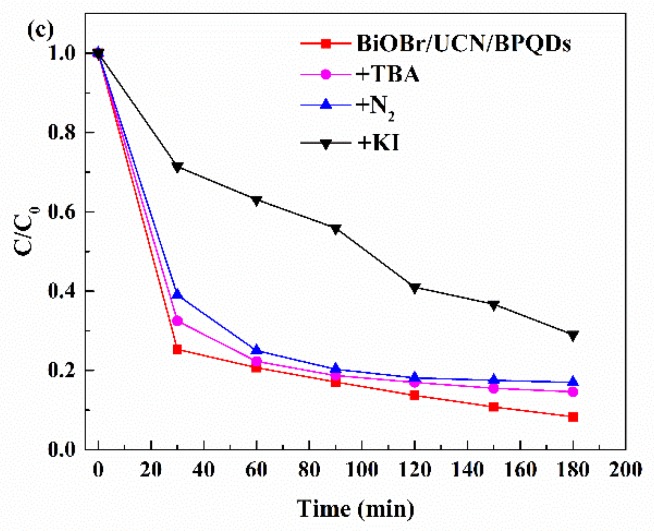
Electron paramagnetic resonance spectra of (**a**) superoxide free radical (·O_2_^−^) in dark and light and (**b**) hole (h^+^) in dark and light. (**c**) Degradation effect after t-butanol (TBA), N_2_, and potassium iodide (KI) catalyst addition.

In order to investigate the specific principles, we tested the electron paramagnetic resonance BiOBr/UCN/BPQDs composites using capture agents for verification. During the photocatalytic process, superoxide free radicals (·O_2_^−^), hydroxyl radicals (·OH^−^), and e^−^–h^+^ pairs are usually involved. Therefore, t-butanol, N_2_, and potassium iodide were used to capture ·OH^−^, ·O_2_^−^, and h^+^, respectively. Electron paramagnetic resonance spectra indicated h^+^ and ·O_2_^−^ were the key groups determining the catalytic degradation effect (Figure 11). Further, following the addition of potassium iodide, the catalytic effect of N_2_ catalyst was significantly reduced [40].

**Figure 12:**
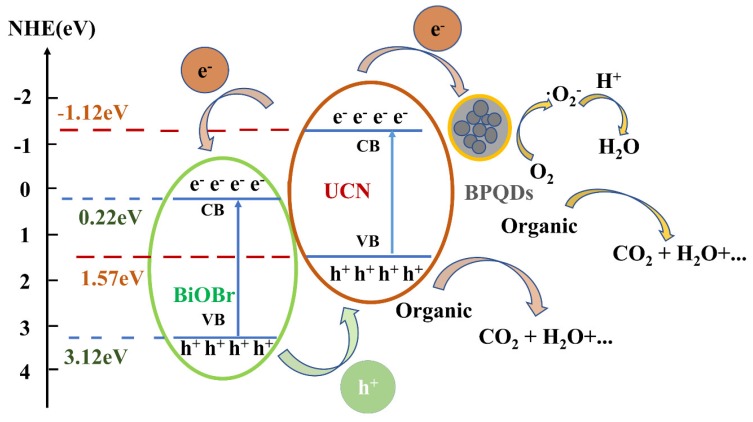
The band structure diagram of tetracycline degradation and possible mechanisms.

The band structure diagram of TC degradation and the possible mechanisms involved are shown in a schematic diagram in Figure 12. The CB and VB of BiOBr were shown to be 0.22 and 3.12 eV, respectively, whereas those of UCN were −1.12 and 1.57 eV, respectively. Because of the particle size relationship between the BPQDs, the band gap hovers between 0.3 and 1.8 eV such that the exact value is not listed here. Nevertheless, it can be determined that this ternary heterogeneous structure forms a bandgap suitable for the generation and separation of photonic carriers, thus greatly enhancing the role of·O_2_^−^·and h^+^ in catalytic degradation. [41,42]

## 4. Conclusions

The BiOBr/UCN/BPQDs tri-heterostructures in the above experiments were prepared by a sonication and hydrothermal method. Compared with BiOBr/UCN, pure g-C_3_N_4_, and pure BiOBr, the prepared composite has a better catalytic degradation efficiency for TC under visible light. The increase in photocatalytic activity may be related to the narrow band gap formed between the heterojunctions, which greatly increases the separation rate of photo-generated carriers and suppresses their recombination. Experimental results showed that superoxide radicals (O_2_^–^) and e^–^–h^+^ pairs are the most active radicals produced during the degradation process. This highly efficient, environmentally friendly, and relatively simple to produce ternary heterostructure has potential applications in the degradation of antibiotics such as TC.
